# Vascular Disease in Systemic Sclerosis

**DOI:** 10.1155/2010/714172

**Published:** 2010-10-26

**Authors:** Lorinda Chung, Oliver Distler, Laura Hummers, Eswar Krishnan, Virginia Steen

**Affiliations:** ^1^Division of Immunology and Rheumatology, Stanford University School of Medicine, Palo Alto VA Health Care System, 3801 Miranda Avenue, Palo Alto, CA 94305, USA; ^2^Department of Rheumatology, University Hospital Zurich, Gloriastr. 25, 8091 Zürich, Switzerland; ^3^Division of Rheumatology, Johns Hopkins University, 5501 Hopkins Bayview Circle, Room 1B.7, Baltimore, MD 21224, USA; ^4^Division of Immunology and Rheumatology, Stanford University School of Medicine, 1000 Welch Road, Suite 203, Palo Alto, CA 94305, USA; ^5^Division of Rheumatology, Georgetown University, 3800 Reservoir Road NW, Pasquerilla Healthcare Center, Suite 3004, Washington, DC 20007, USA

Systemic sclerosis (SSc) is an autoimmune disease characterized by widespread fibrosis affecting the skin, internal organs, and vasculature. Vascular disease with intimal proliferation and obliterative vasculopathy is extremely prevalent in SSc, most commonly manifesting as Raynaud's phenomenon and digital ulcerations. Other manifestations of vascular disease occur less frequently in patients with SSc, including ischemic digital loss, pulmonary arterial hypertension (PAH), and renal crisis. Vascular disease in SSc may also commonly affect pregnancy outcomes and sexual function. Finally, patients with SSc can rarely suffer from inflammatory vasculitis, and recognition of this has a significant impact on therapy. In this special issue on vascular disease in systemic sclerosis, we invited original research articles and review articles regarding the pathogenesis, epidemiology, natural history, evaluation, and/or management of vascular complications in patients with SSc.

The first paper of this special issue describes the vascular microenvironment that likely contributes to the pathogenesis of vasculopathy in SSc. This is followed by two papers of the current knowledge regarding the pathogenesis, evaluation, and management of digital ulcers and digital ischemic loss in patients with SSc. The fourth paper of the special issue is an original research article that describes the prevalence of and risk factors for nondigital lower extremity ulcers in SSc. The authors found that antiphospholipid antibodies and genetic prothrombotic mutations are highly prevalent in SSc patients with lower extremity ulcers. The next paper describes the utility of registries in understanding the natural history of digital ulcers and discusses the need for classification criteria for the assessment of digital ulcers. This is followed by a review article discussing the potential use of nailfold videocapillaroscopy as an outcome measure in clinical trials for patients with SSc and vascular complications, particularly digital ulcers and PAH.

The next section of the special issue relates to cardiopulmonary vascular complications in SSc, with a particular focus on PAH and right ventricular failure. The first paper in this section describes a case report of a patient with mixed connective tissue disease and severe, refractory PAH who experienced dramatic improvement in functional ability and hemodynamics in response to treatment with tocilizumab, a humanized monoclonal antibody to the human interleukin-6 receptor. This is followed by an original research article evaluating the relationship of serum endoglin levels in patients with and without elevated systolic pulmonary arterial pressures (sPAP) on echocardiography. This study found that SSc patients with and without elevated sPAP had much higher levels of serum endoglin compared with healthy controls, suggesting that endoglin may be a potential biomarker of vasculopathy that is not specific to the pulmonary vasculature. The next paper describes a histopathogic comparison of samples from the right ventricle of patients with SSc-associated PAH and idiopathic PAH (IPAH). The authors found that the right ventricular samples from patients with SSc-associated PAH showed more inflammatory infiltrates than those from patients with IPAH, but the degree of interstitial fibrosis was similar in the two groups. The final paper in this section is a review describing available nuclear cardiology imaging modalities that may be useful in the assessment of early vascular disease and myocardial damage in patients with SSc.

The next section of the special issue is focused on renal manifestations of SSc. The first paper reviews the types of renal involvement seen in SSc and the potential utility of screening patients for subclinical renal disease. The next paper describes the histopathologic findings characteristic of scleroderma renal crisis (SRC) and the potential role of renal biopsies in predicting prognosis. Finally, an original research article describes the methodology and preliminary data of an international web-based prospective study designed to determine if the use of angiotensin converting enzyme inhibitors prior to the onset of SRC is associated with worse outcomes.

The subsequent section of the special issue addresses the effects of vascular disease on pregnancy and sexual function in patients with SSc. The first paper reviews the published literature regarding the effects of vascular complications, such as PAH and SRC, on pregnancy outcomes in patients with SSc. This is followed by review articles focusing on sexual dysfunction in patients with SSc, including male erectile dysfunction and female sexual arousal disorder. These papers also address the role of phosphodiesterase-5 inhibitors in the treatment of SSc patients with sexual dysfunction.

The final section includes two papers describing the known data on inflammatory vasculitis in patients with SSc. The first focuses on microscopic polyangiitis, while the latter article reviews the literature regarding small, medium, and large vessel vasculitis in SSc.

In summary, this special issue provides a comprehensive compilation of articles with novel information relevant to the topic of vascular disease in SSc. The papers included herein provide the framework for the current and future development of important biomarkers, outcome assessments for clinical trials, and novel therapeutic modalities for patients with SSc and vascular complications. We, the guest editors, hope that readers find this information useful in the care of patients with SSc.

*Lorinda Chung* *Lorinda Chung* *Oliver Distler* *Oliver Distler* *Laura Hummers* *Laura Hummers* *Eswar Krishnan* *Eswar Krishnan* *Virginia Steen* *Virginia Steen*

